# Early identification and integrated nursing management of post-thrombolysis hemorrhagic transformation in acute ischemic stroke: a comprehensive review

**DOI:** 10.3389/fneur.2025.1704431

**Published:** 2026-01-07

**Authors:** Liu Yang, Chao Hu, Qiucen Yang, Hua Huang, Xuan Hou

**Affiliations:** Dazhou Central Hospital, Dazhou, China

**Keywords:** hemorrhagic transformation, nursing management, risk factors, stroke, thrombolytic therapy

## Abstract

Acute ischemic stroke (AIS) is a leading cause of high global disability and mortality rates. Intravenous thrombolysis (IVT) is a key therapeutic intervention for restoring blood flow and salvaging the ischemic penumbra. However, it can be complicated by severe hemorrhagic transformation (HT). Symptomatic intracranial hemorrhagic transformation significantly increases the risk of death and disability in patients and is a major limiting factor affecting the benefit of thrombolysis. This article aims to comprehensively analyze relevant literature and systematically explore early identification methods for hemorrhagic transformation following thrombolysis in AIS patients, as well as scientific and systematic nursing management strategies. By reviewing key risk factors, pathological mechanisms, and evidence-based interventions, it seeks to provide a reference basis for the clinical early identification of patients at high risk for HT, the implementation of targeted nursing interventions to mitigate the harm of HT, and the improvement of patient outcomes.

## 1 Introduction

Acute Ischemic Stroke (AIS), as the predominant type of cerebrovascular disease, accounts for 60%–80% of all stroke cases (1). Its core pathology lies in the sudden blockage of cerebral blood vessels leading to interruption of blood flow to brain tissue. It is characterized by extremely rapid onset, swift progression, and persistently high rates of disability and mortality. Epidemiological data indicate that AIS is not only the leading cause of death among Chinese residents but also the primary factor causing long-term disability in adults, imposing a substantial burden on patients, families, and society (2). Within the AIS treatment system, early intravenous thrombolytic therapy (such as rt-PA) is a critical, time-sensitive treatment strategy aimed at restoring blood flow and salvaging the ischemic penumbra – brain tissue that is functionally impaired but not yet irreversibly infarcted (3). Its core mechanism involves dissolving the occluding thrombus to achieve recanalization of the responsible blood vessel, thereby maximizing the preservation of neurological function. However, this therapy carries the risk of inducing HT. HT refers to the occurrence of bleeding or hematoma formation within the ischemic brain tissue following the restoration of blood flow. Symptomatic HT, once it occurs, can lead to a worsening of the patient’s neurological deficits. In severe cases, it can cause significant intracranial hypertension, brain herniation, and even life-threatening complications (4). Therefore, the early and precise identification of patient populations at high risk for HT and the timely implementation of scientific and systematic nursing management strategies are crucial.

## 2 Pathogenesis of HT post-thrombolysis

A deep understanding of the mechanisms underlying HT is fundamental for its early identification and effective intervention. Currently, the pathogenesis of HT following thrombolysis remains incompletely elucidated and is considered a complex pathophysiological cascade resulting from the interaction of multiple factors and processes. Research consensus primarily focuses on the following core mechanisms: endothelial injury after recanalization, ischemia–reperfusion injury and oxidative stress storm, malignant amplification of the inflammatory response and mediator release, and severe disruption of the blood–brain barrier (BBB) (5–8).

While successful vessel recanalization and blood flow restoration are the therapeutic goals, the sudden restoration of flow itself constitutes a mechanical injury to the fragile vascular bed already compromised by severe ischemia. More critically, during ischemia, vascular endothelial cells suffer from energy metabolism failure and impaired structural integrity. Upon reperfusion, the shear stress of high-velocity blood flow acts directly on the dysfunctional endothelium (9, 10), leading to the destruction of tight junctions between endothelial cells, disassembly of adherens junctions, disruption of endothelial continuity, and a dramatic increase in vascular permeability. This allows plasma components and even red blood cells (RBCs) to extravasate, forming the initial pathological basis for hemorrhage. During the period of interrupted blood flow, energy is depleted within the tissue, ion pumps fail, and calcium overload occurs (11). Once blood flow is restored and oxygen is resupplied, it paradoxically triggers a series of intense biochemical reactions. Cells with mitochondrial dysfunction generate large amounts of reactive oxygen species (ROS), such as superoxide anion, hydrogen peroxide, and hydroxyl radicals (12). These highly reactive molecules overwhelm the scavenging capacity of endogenous antioxidant systems (e.g., superoxide dismutase (SOD), glutathione), initiating an oxidative stress storm. ROS indiscriminately attack cell membrane phospholipids, proteins, and DNA, directly causing irreversible damage and death to key BBB-forming cells, including endothelial cells, astrocytes, and pericytes, further undermining vascular structural stability (13). Ischemia itself initiates the innate immune response. Reperfusion significantly amplifies the inflammatory cascade. Damaged brain tissue releases a large number of damage-associated molecular patterns (DAMPs) (14, 15), activating microglia and infiltrating inflammatory cells such as neutrophils and monocytes/macrophages. These hyperactivated cells release copious amounts of pro-inflammatory cytokines (e.g., TNF-α, IL-1β, IL-6) and chemokines (e.g., MCP-1, IL-8), creating a vicious cycle of inflammation (16, 17). More importantly, activated neutrophils release elastase, myeloperoxidase (MPO), and particularly matrix metalloproteinases (MMPs, especially MMP-9) (18). MMP-9 efficiently degrades key components of the vascular basement membrane and extracellular matrix, such as type IV collagen, laminin, and fibronectin, causing severe disruption to the structural integrity of the BBB (19, 20).

The final common pathway of endothelial injury, oxidative stress, and inflammatory attack is severe disruption of the BBB (21). An intact BBB relies on a sophisticated structure: endothelial cells with their tight junctions, an intact basement membrane, and the coverage provided by astrocytic end-feet and pericytes. The mechanisms described above act synergistically (19, 22), leading to degradation of tight junction proteins, disintegration of the basement membrane, pericyte contraction or detachment, and astrocyte dysfunction. The resultant extreme increase in BBB permeability allows plasma proteins, inflammatory cells, and especially RBCs—normally strictly excluded from the brain parenchyma—to freely extravasate into the brain interstitial space. Hemoglobin and its degradation products (e.g., iron ions) released from ruptured RBCs possess potent neurotoxicity and pro-inflammatory, pro-oxidant effects, causing secondary injury. This further exacerbates tissue edema and neuronal damage, becoming visible as hemorrhagic foci on imaging. These manifest as either petechial hemorrhages (Hemorrhagic Infarction, HI types HI-1/HI-2) or dense hematomas (Parenchymal Hematoma, PH types PH-1/PH-2) (23, 24).

## 3 Risk factors for HT post-thrombolysis

The occurrence of HT after thrombolysis results from the interplay of multiple risk factors, including the patient’s baseline condition, stroke severity, and characteristics of the therapeutic intervention. A deep understanding of these factors is crucial for accurate risk assessment, optimizing patient selection, and implementing individualized monitoring.

Patient-related risk factors constitute the foundation of intrinsic susceptibility. Advanced age (especially >80 years) is one of the most significant independent predictors. This stems from the fragility of the cerebrovascular structure in elderly patients (e.g., atherosclerosis, cerebral amyloid angiopathy), diminished coagulation function, and reduced endothelial repair capacity (25). Gender differences have also been noted. Relevant studies (26) suggest that female patients may have a slightly higher risk of symptomatic intracranial hemorrhage than males (OR ≈ 1.3). Potential mechanisms may involve differences in hormone levels, cerebrovascular anatomy, or drug metabolism. Severity of cerebral infarction is a core clinical indicator. A higher baseline National Institutes of Health Stroke Scale (NIHSS) score indicates a larger infarct core volume, greater fragility of the ischemic penumbra tissue, and more severe impairment of cerebrovascular autoregulation, significantly increasing the risk of hemorrhage upon reperfusion (27). Atrial fibrillation (AF), as the primary cause of cardioembolic stroke, often leads to large vessel occlusion by large thrombi rich in fibrin/red blood cells, resulting in large infarcts. Furthermore, atrial blood stasis, endothelial damage, and the frequently associated hypercoagulable/anticoagulant background make the vascular bed more susceptible to bleeding triggered by the recanalization shock of thrombolysis (28). Hyperglycemia is a well-established independent risk factor (29). It compromises BBB stability through multiple pathways, including exacerbating oxidative stress, impairing endothelial function, promoting inflammation, and worsening acidosis. Prior history of stroke reflects poorer underlying cerebrovascular conditions, often involving chronic cerebral small vessel disease, abnormal vascular bed structure, or uncontrolled vascular risk factors. This significantly weakens vascular reserve and repair capacity (30).

Treatment-related risk factors focus on the choice and timing of the intervention itself. The dose and type of thrombolytic agent directly impact risk. Strict adherence to weight-based dosing regimens (e.g., rt-PA 0.9 mg/kg, maximum 90 mg) is paramount; overdose is an absolute contraindication. Different agents have distinct fibrinolytic properties and safety profiles. Newer agents like tenecteplase, due to their higher fibrin specificity (31) and longer half-life (32), show potential safety advantages, but definitive evidence for reducing HT requires further study. The thrombolysis time window is the cornerstone of safety. The current intravenous rt-PA window is strictly limited to within 4.5 h of symptom onset. Thrombolysis beyond this window dramatically increases risk because, with delayed treatment, brain tissue damage causes infarct core expansion and penumbra loss, while vascular endothelium and BBB disruption are extremely severe or even necrotic. Restoring blood flow at this stage is highly likely to cause severe hemorrhagic consequences (33, 34).

Other critical risk factors must not be overlooked. Uncontrolled hypertension prior to thrombolysis (especially SBP > 180 mmHg or DBP > 110 mmHg) is a contraindication or requires urgent management. Severe blood pressure fluctuations or sustained hypertension after thrombolysis is a direct mechanical factor inducing HT (35). Early imaging changes (36–38) are strong predictors. Findings such as large hypodense areas on CT (>1/3 MCA territory), significant mass effect, low ASPECTS score (<7), or large diffusion-restricted volume on MRI-DWI all indicate severe infarction and a very high risk of HT. Pre-thrombolytic use of antiplatelet or anticoagulant medications significantly increases bleeding risk. Thrombolysis with an INR > 1.7 is associated with an sICH risk as high as 12.5% (vs. 4.1% without anticoagulation, *p* < 0.001) (39). Renal insufficiency may affect drug clearance, increasing exposure risk.

Therefore, HT risk is the result of interactions between patient baseline status (age, gender, stroke history, cerebrovascular disease), current stroke characteristics (severity/NIHSS, AF, hyperglycemia), therapeutic intervention (drug type and dose, time window management), and other factors (blood pressure, imaging findings, concomitant medications). Clinical practice necessitates comprehensive, individualized risk assessment, strict patient selection, precise medication administration, rigorous control of the time window and blood pressure, and implementation of intensive post-thrombolysis monitoring to maximize the benefit of thrombolysis while minimizing hemorrhagic consequences (Table 1).

**Table 1:** Key risk factors for hemorrhagic transformation post-thrombolysis.

Category	Risk factor	Key considerations/clinical implications
Patient-related	Advanced age (esp. >80 years)	Fragile cerebrovascular structure, reduced repair capacity.
	Severe stroke (High NIHSS score)	Larger infarct core, more compromised penumbra, impaired autoregulation.
	Atrial fibrillation	Often leads to large, cardioembolic infarcts with higher HT risk.
	Hyperglycemia	Exacerbates oxidative stress, inflammation, and BBB disruption.
	Prior stroke/cerebral small vessel disease	Indicates poor baseline cerebrovascular health and reserve.
Treatment-related	Thrombolytic agent dose and type	Overdose (e.g., rt-PA > 0.9 mg/kg) increases risk. Higher fibrin specificity (e.g., Tenecteplase) may offer safety benefits.
	Delayed time to thrombolysis (>4.5 h)	Increased likelihood of irreversible BBB disruption and tissue necrosis.
Other modifiable factors	Uncontrolled hypertension (pre or post-thrombolysis)	High SBP/DBP is a direct mechanical risk for vessel rupture.
	Early ischemic changes on imaging	CT: low ASPECTS, large hypodensity (>1/3 MCA) indicates extensive infarction.
	Concomitant use of anticoagulants/antiplatelets	Significantly elevates bleeding risk, especially with elevated INR.

## 4 Methods for early identification of HT post-thrombolysis

Early identification of HT post-thrombolysis is crucial for improving patient outcomes and requires establishing a dynamic monitoring framework integrating a “tripartite approach: clinical assessment—imaging examination—biomarker analysis.” Imaging Techniques: Imaging is central to diagnosing structural damage (40, 41).

Computed Tomography (CT) Scan: Serves as the first-line screening tool. It can rapidly confirm Parenchymal Hematoma (PH) or Hemorrhagic Infarction (HI) types. Dynamic repeat scans within 24 h post-thrombolysis (especially during the 2 to 6-h high-risk period) are essential to detect new-onset hemorrhage, expansion of the bleed, and mass effect. However, its sensitivity is limited for microbleeds (<5 mm) and pathologies in the posterior fossa. Magnetic Resonance Imaging (MRI) provides precise complementary information through its multi-sequence capabilities. Gradient-Recalled Echo (GRE) or Susceptibility-Weighted Imaging (SWI) highly sensitive for detecting microbleeds and early petechial hemorrhage. Diffusion-Weighted Imaging (DWI) defines the extent of the infarct core. Fluid-Attenuated Inversion Recovery (FLAIR) helps differentiate hemorrhage from edema. Dynamic Contrast-Enhanced MRI (DCE-MRI) can quantitatively assess the degree of blood–brain barrier (BBB) disruption, providing very early warning of HT risk.

Quantitative Neurological Function Assessment (42): Forms the cornerstone for detecting clinical deterioration. NIHSS remains the gold standard. Intensive monitoring within 24 h post-thrombolysis (e.g., hourly) is mandatory. An acute increase in NIHSS score of ≥4 points or the emergence of new focal neurological signs (e.g., decreased level of consciousness, anisocoria, worsening hemiparesis) should trigger immediate imaging evaluation. Other scales like the European Stroke Scale (ESS) can serve as supplementary tools, but the NIHSS remains irreplaceable as the core assessment metric.

Biomarkers, Provide early warning signals at the molecular level reflecting the internal milieu (43–45). Dynamic monitoring of D-dimer offers the highest clinical value. An abrupt, steep rise or persistently very high levels 2–6 h post-thrombolysis indicates excessive fibrinolysis and signals an elevated HT risk. Other potential biomarkers, while still under investigation, may enhance predictive power when analyzed in combination. Among these, a panel combining D-dimer, MMP-9, and GFAP appears particularly promising for early risk stratification, reflecting ongoing fibrinolysis, blood–brain barrier disruption, and astroglial injury, respectively.

Therefore, the real-time cross-validation of multimodal data (clinical, imaging, biomarker) enables the early prediction of HT, providing a critical time window for interventions aimed at salvaging neurological function.

## 5 Nursing management strategies for HT post-thrombolysis

For patients experiencing HT following thrombolysis for AIS, implementing scientific, comprehensive, and dynamic nursing management is crucial. The primary goals are early risk identification, stabilization of vital signs, prevention of complications, provision of psychological support, and promotion of functional recovery.

Strengthened Condition Monitoring and Vital Sign Management form the cornerstone of nursing care. Nurses must maintain high vigilance through close bedside monitoring. Assessments every 15–30 min should systematically evaluate consciousness level, pupillary changes, and critical vital signs, particularly abnormal fluctuations in blood pressure, heart rate, and respiration, which often serve as early warning signs of deterioration. Regular neurological assessments, such as the NIHSS score, effectively quantify changes in neurological deficits. Simultaneously, heightened attention must be paid to patient complaints, especially worsening symptoms like headache, nausea, and vomiting. Sudden severe headache or frequent vomiting necessitates immediate physician notification and assistance with investigations like cranial CT scans to rule out increased intracranial pressure (ICP) or worsening hemorrhage. Within vital sign management, meticulous blood pressure control is paramount. Individualized targets should be set based on the patient’s age, baseline blood pressure, and vascular pathology. Priority should be given to continuous intravenous infusion of antihypertensive agents (e.g., Urapidil, Nicardipine) *via* syringe pump to ensure gradual and controlled reduction, avoiding precipitous drops that could cause cerebral hypoperfusion or excessively high levels that exacerbate bleeding. Maintaining a patent airway is another critical task. This involves prompt clearance of secretions. Patients with thick sputum require nebulization and suctioning. For those with weak spontaneous respirations or severe impairment of consciousness, rapid assistance with endotracheal intubation is essential to ensure adequate oxygenation and prevent hypoxic brain injury. Furthermore, nurses play a critical role in the biospecimen monitoring pipeline. They are responsible for ensuring the timely collection and dispatch of blood samples for biomarker assays (e.g., D-dimer, MMP-9). They must also be vigilant in tracking the results, recognizing abnormal trends such as a sharp rise in D-dimer, and communicating these findings promptly to the physician team to facilitate early intervention.

Proactive Prevention and Management of Complications is key to improving prognosis. Nursing interventions must employ multiple strategies to reduce complication risks. To mitigate the risk of increased ICP, elevate the head of the bed 15°–30° to promote venous drainage and reduce cerebral edema, while closely observing for associated symptoms and responses to dehydrating agents. Infection prevention is of utmost importance and includes: strict adherence to twice-daily oral care; regular repositioning (at least every 2 h) with maintenance of skin cleanliness and dryness to prevent pressure injuries; and for patients with indwelling urinary catheters, enhanced perineal care following strict aseptic technique and scheduled catheter/bag changes to prevent urinary tract infections. Antibiotic use must strictly follow medical orders, ensuring precise selection and timely administration to avoid misuse that could lead to flora imbalance and secondary infections. For long-term bedridden patients, prevention of deep vein thrombosis (DVT) and pulmonary embolism (PE) is a major focus. Encourage early passive limb exercises and utilize adjunctive measures like compression stockings or intermittent pneumatic compression devices to promote lower limb venous return.

Providing Comprehensive Psychological Support and Early Rehabilitation Intervention are indispensable components for promoting holistic patient recovery. Patients and their families often experience significant anxiety and fear. Healthcare providers should proactively communicate, explaining the condition and treatment plan in understandable language, listening patiently to concerns and questions, and offering emotional support and reassurance. Sharing success stories or organizing peer support groups for similar patients can help build confidence and foster a positive attitude towards the illness. Once the patient’s condition stabilizes, the rehabilitation nursing team should intervene early. Develop individualized, stepwise rehabilitation plans based on the degree of neurological deficit and overall physical status. Training content should encompass: limb function, language function and cognitive function. Nurses must closely monitor patient responses and training effectiveness, adjusting plans promptly to ensure safety and efficacy. Concurrently, reinforce rehabilitation education and skill instruction for patients and familie to enhance adherence and self-management capabilities post-discharge. The ultimate goal is to promote functional recovery, improve self-care ability, and facilitate successful reintegration into society.

Nursing management for post-thrombolysis HT patients constitutes a multidimensional, dynamic intervention system integrating rigorous monitoring, precise treatment, complication prevention and control, psychological care, and scientific rehabilitation. Each component is interlinked and mutually reinforcing, requiring collaborative execution by a multidisciplinary team. Implementing this comprehensive, meticulous, and personalized nursing strategy is essential to maximize patient safety, optimize neurological recovery, and improve long-term outcomes.

## 6 Future perspectives

Advances in medical technology are expected to yield further breakthroughs. Enhanced imaging modalities—such as high-resolution MRI and functional MRI (46, 47)—will provide clearer and more precise visualization of cerebrovascular structures and brain tissue, enabling earlier detection of HT signs. Biomarker research will deepen through large-scale clinical studies, aiming to identify combinations with superior specificity and sensitivity. This may facilitate predictive model development to improve HT risk stratification post-thrombolysis.

In nursing interventions, intelligent monitoring systems (48) (e.g., non-invasive continuous cerebral edema/intracranial pressure monitoring) show promise for earlier warning capabilities. Concurrently, evidence-based standardized nursing pathways warrant development and validation as key directions for enhancing care efficacy. Strengthened specialized training for nursing staff remains crucial. This will elevate proficiency in HT recognition and management while fostering more effective nursing interventions. Such advancements will substantially support rehabilitation and quality-of-life improvements in acute ischemic stroke patients.

In summary, thrombolytic therapy for AIS requires careful risk–benefit assessment. While pursuing timely reperfusion, comprehensive strategies for early HT identification and meticulous nursing management must be implemented to mitigate risks. Continued medical innovations will bring renewed hope to this field, ultimately saving more lives threatened by stroke.
